# Rapid On-Site Detection of *Pseudomonas aeruginosa* via ecfX-Targeted Loop-Mediated Isothermal Amplification

**DOI:** 10.3390/bios15110750

**Published:** 2025-11-07

**Authors:** Xuliang He, Meimei Zeng, Wentao Bai, Ziyan Tang, Jianhua Ding, Zhu Chen

**Affiliations:** 1 Department of General Surgery, The Affiliated Zhuzhou People’s Hospital of Changsha Medical University, Zhuzhou 412000, China; 2 Hunan Key Laboratory of Biomedical Nanomaterials and Devices, School of Biological Science and Medical Engineering, Hunan University of Technology, Zhuzhou 412007, China; 3 MOE Key Lab of Rare Pediatric Diseases & Hengyang Medical College, Hengyang Medical School, University of South China, Hengyang 421001, China; 4 Institute for Future Sciences, University of South China, Changsha 410008, China; 5 Department of Surgery, The Affiliated Zhuzhou People’s Hospital of Changsha Medical University, Zhuzhou 412000, China

**Keywords:** *Pseudomonas aeruginosa*, fluorescence detection, loop-mediated isothermal amplification, ecfX gene

## Abstract

*Pseudomonas aeruginosa* (PA) is a significant pathogen of clinical concern that is frequently associated with multidrug resistance, leading to respiratory tract, wound, and hospital-acquired infections. To enable rapid and accurate detection, we developed a fluorescence-based loop-mediated isothermal amplification (LAMP) method, targeting the PA-specific ecfX gene. Among ten primer sets designed, the optimal set (EC2) was identified, and reaction conditions were optimized (Bst polymerase 320 U/mL, Mg^2+^ 8 mM, dNTP 1.4 mM, inner/outer primer ratio 1:8, 64 °C, 20 min). The assay demonstrated a detection limit that was comparable to a real-time polymerase chain reaction and immunochromatographic assays, but with a markedly reduced turnaround time. No cross-reactivity was observed with non-PA pathogens, and reproducibility tests confirmed high stability. In addition, the reliability of the results was further verified using 60 standard bacterial strains, and the feasibility of the assay was validated with 2 real soil samples and 1 water sample. This LAMP method offers a simple, rapid, and sensitive tool for on-site detection of PA, with potential applications in clinical diagnostics and public health surveillance.

## 1. Introduction

*Pseudomonas aeruginosa* (PA) frequently exhibits multidrug resistance and is a major cause of respiratory tract infections, wound infections, and nosocomial infections in intensive care units. Early and accurate identification of PA is of great clinical significance for guiding rational antibiotic use and preventing the spread of infection [1]. Traditional methods such as bacterial culture and biochemical identification are time-consuming and show limited sensitivity, making them insufficient for rapid emergency detection [2]. The ecfX genesis is a highly conserved and specific target that is closely associated with virulence factors and has been demonstrated to possess higher specificity than commonly used markers such as the outer membrane protein genes oprI, oprL, and 16S rRNA [3]. Consequently, it represents an ideal molecular target for the detection of *P. aeruginosa*. Nucleic acid assays based on ecfX are not only suitable for clinical specimens but also perform well in complex matrices such as water and environmental samples, making them particularly advantageous for on-site rapid screening and environmental health surveillance. Such assays contribute to early warnings and source tracking of PA during public health emergencies [4].

Currently, quantitative polymerase chain reaction (qPCR) assays targeting the ecfX gene have been widely applied for the rapid identification of PA. This method enables amplification within a few hours, offering high sensitivity and accuracy, thereby providing reliable evidence for clinical diagnosis [5]. However, the requirement for sophisticated laboratory equipment limits its application in resource-limited or field settings. In contrast, loop-mediated isothermal amplification (LAMP) is a highly efficient nucleic acid amplification technique performed under constant temperature conditions. It utilizes a DNA polymerase with strand-displacement activity (such as Bst) at 60–65°C to rapidly replicate the target sequence. The reaction system typically consists of four to six specific primers that recognize six different regions of the target gene, ensuring high specificity [6]. During the LAMP reaction, internal and external primers work together to generate a dumbbell-shaped DNA template. Circular strand displacement reactions then form a large number of circular amplification products, achieving amplification efficiencies as high as 10^9^-fold within 30–60 min. Detection can be achieved through fluorescence, color change, or turbidity changes, providing visualization without the need for expensive instrumentation. This method is widely used for rapid on-site pathogen detection and molecular diagnostics [7]. This method has certain limitations in terms of quantitative accuracy compared to qPCR. Although LAMP has the advantages of simple operation and fast speed, which makes it suitable for point-of-care testing on-site testing, its reaction kinetics differences, reagent stability, and the subjectivity of color interpretation may affect the consistency of the results. To improve reliability, we must undertake further exploration of signal quantification strategies such as real-time LAMP. Through the analysis of qPCR amplification curves and the correlation of detection results, the reproducibility and quantitative consistency of the LAMP system in clinical sample testing can be verified [8]. Liu et al. developed a rapid detection system based on RPA/CRISPR/Cas12a targeting the oprL gene of PA, with a detection limit of 60 fg/reaction and 100% specificity. Federica et al. investigated the use of LAMP for the detection of three pathogens on hospital surfaces and compared it with the traditional gold standard culture method. The detection limit for PA was 1.5 × 10^2^ CFU/mL in 9 h. In contrast, no colonies were detected in the culture method at 1.5 × 10^1^ CFU/mL or in the negative control [9]. Ferrusca Bernal et al. developed a detection system combining LAMP with reverse line hybridization (RLBH) for a specific region of the PA 16S rRNA gene. The sensitivity of gel electrophoresis imaging was 3 × 10^−4^ ng/µL, demonstrating good specificity [10]. Qiu et al. developed a single-tube reaction system integrating LAMP and CRISPR-Cas, enabling a single-step, fully closed reaction. The detection limit was 10 copies per reaction system, and the specificity of the test for 48 strains was 100% [11].

Moreover, Alhogail et al. proposed a rapid visual detection platform based on magnetic nanoparticles and colorimetric signals for the LasA protease activity that was specific to PA. This platform achieves a highly specific and low-cost identification of *P. aeruginosa* within 1 min, with a detection sensitivity of 10^2^ CFU/mL and a detection time of less than 1 min. It is suitable for point-of-care diagnostics. However, its limitations lie in its reliance on live bacterial protease activity, lack of quantitative accuracy, and extensive clinical validation [12]. Khan et al. described an electrochemical sensor for detecting phenazine metabolites (such as pyocyanin (PYO)) secreted by *P. aeruginosa*. The detection limit can reach 10^2^–10^3^ CFU/mL, with a detection time of 30–60 min. However, while PYO is a characteristic metabolite of *P. aeruginosa*, other bacterial metabolites (such as flavins and quinones) can also generate redox signals, causing background interference [13]. Furthermore, standardized signal calibration and quantitative models for multi-bacterial coexistence are lacking. Thus, despite the rapidity of electrochemical or colorimetric detection, specificity and quantitative accuracy are limited. The LAMP-based method has very high detection speed, sensitivity, and specificity, with its amplification speed being 1000 times that of PCR. Through correlation analysis with fluorescence quantitative PCR curves, it has the potential to achieve quantitative detection [14].

Therefore, this study designed an optimal set of LAMP primers targeting the ecfX gene and established and optimized a rapid detection system for PA based on LAMP. The feasibility of this method was verified using fluorescence detection, gel imaging, and test strips. The goal is to provide an efficient, sensitive, and field-ready pathogen detection method, providing solid technical support for the precise diagnosis and public health prevention and control of PA infection.

## 2. Materials and Methods

### 2.1. Reagents and Materials

The Mag-MK Bacterial Genomic DNA Extraction Kit (B518725-0050, Sangon Biotech, Shanghai, China) was used for genomic DNA extraction. Bst II Pro DNA Polymerase Large Fragment (P703-01, Vazyme, Nanjing, China), dNTPs and LAMP Fluorescent Dye (New England Biolabs, Ipswich, MA, USA) were employed for the LAMP assay. Goldview Nucleic Acid Gel Stain (10,000×, Yeasen Biotechnology, Shanghai, China), agarose, 6× DNA loading buffer, DNA marker (100–600 bp), ddH_2_O, 50× TAE buffer, and primers were purchased from Sangon Biotech. The probe-based real-time PCR kit for *P. aeruginosa* was obtained from Hangzhou Geju Medical Technology Co., Ltd. (Hangzhou, China), while the lateral flow assay (LFA) strips for nucleic acid amplification product detection were supplied by Jieshi East Biotech (Xiamen, China). All bacterial strains used in this study were kindly provided by Zhuzhou People’s Hospital.

### 2.2. Bacterial Culture and DNA Extractions

PA, *Mycobacterium tuberculosis*, *Helicobacter pylori*, *Escherichia coli*, and *Staphylococcus aureus* were included in this study. Each strain was cultured under its respective optimal growth conditions, following standard microbiological protocols. Genomic DNA was extracted from the cultured strains using the commercial kit, according to the manufacturer’s instructions.

The extracted DNA was subsequently used to assess the specificity of the developed LAMP assay, with PA serving as the target strain and the other bacterial species as non-target controls. All strains were preserved in the laboratory for experimental validation throughout this study.

The specific process of rapid testing of water or soil environmental samples is as follows.

For PA detection in water samples:

(1). Collect at least 500 mL to 1000 mL of the water sample in a sterile bottle (more water may be required if the bacterial count is low). 

(2). Use a syringe to push a certain volume of the water sample (e.g., 100 mL to 500 mL) through the filter membrane or vacuum filter.

(3). Remove the filter membrane and place it in a sterile centrifuge tube. Add 1–2 mL of sterile eluent (e.g., PBS) and vortex vigorously to elute the bacteria from the membrane.

For PA detection in soil samples:

(1). Use sterile tools to collect representative soil samples from different locations and mix thoroughly.

(2). Weigh a certain amount of soil (e.g., 1 g) and add it to a sterile buffer (e.g., PBS or saline), typically at a ratio of 1:10 (e.g., 1 g of soil to 9 mL of liquid).

(3). Vigorously vortex (e.g., vortex for 10–15 min) to dislodge the microorganisms from the soil particles. After standing or low-speed centrifugation (4000 rpm, 5 min) to remove the supernatant, high-speed centrifugation can be performed (such as 12,000 rpm, 10 min), the supernatant can be discarded, and the bacterial pellet can be resuspended in a small amount of buffer.

### 2.3. Fluorescence Result Judgment Standards

A positive result is defined by an S-shape amplification curve exhibiting a distinct exponential growth phase. A negative result is characterized by the absence of a Ct (cycle threshold) in qPCR or TTR (time to result) in qLAMP, displaying either a straight line or slightly inclined curve without an exponential growth phase. TTR value is similar to the PCR cycle number, where the fluorescence signal first crosses the detection threshold. In the qLAMP assay, amplification was performed for 60 min, with fluorescence recorded at 1 min intervals per cycle.

### 2.4. LAMP Primer Design

Based on the ecfX gene sequence of PA registered in GenBank (accession number: CP025055.2), ten sets of LAMP primers were designed using Primer Explorer 5.0 (https://primerexplorer.eiken.co.jp/e/ (accessed on 7 July 2025)). Each set comprised six primers: F3/B3 (outer primers), FIP/BIP (inner primers), and LF/LB (loop primers). After sequence alignment and preliminary evaluation, five primer sets were selected and tested using a standard LAMP reaction system. Primer performance was assessed by both fluorescence amplification curves and agarose gel electrophoresis. The primer set exhibiting the highest specificity and sensitivity was chosen for subsequent experiments.

BLAST (Snapgene 6.0.2) comparison of the EC2 gene with the NCBI genome revealed that the absolutely conserved sequences are B3, FIP, BIP, LF, and LB; the only non-conserved sequence is F3. Although F3 is the only non-conserved sequence in the primer set, F3 and B3 serve as strand displacement anchors, and the specificity of the amplification product is primarily determined by FIP and BIP. Therefore, the specificity of this primer set meets the requirements (see Supplementary Materials). This further demonstrates that the sequence EC2, selected in this study, has high specificity.

### 2.5. Optimization of LAMP Reaction System

To establish the optimal LAMP assay conditions for PA detection, the key reaction parameters were systematically optimized. These included the following: Bst polymerase concentration—160, 240, 320, 400, and 480 U/mL, Mg^2+^ concentration—6, 7, 8, 9, and 10 mM, dNTP concentration—1.2, 1.4, 1.6, 1.8, and 2.0 mM, inner/outer primer ratio—1:1, 1:2, 1:4, 1:6, 1:8, 1:10, and 1:12, reaction temperature: 63, 64, 65, 66, 67, and 68 °C, and reaction time—15, 20, 30, 45, and 60 min. For all reactions, 2 μL of DNA template was used, and a no-template control (NTC) was included for each condition. The performance of each reaction condition was evaluated based on the fluorescence amplification curves and agarose gel electrophoresis, and the combination showing the highest sensitivity and specificity was selected for subsequent experiments.

### 2.6. Sensitivity Assays

PA DNA was measured using a NanoDrop micro-spectrophotometer (Thermo Fisher Scientific, Wilmington, DE, USA). Ds DNA was selected and calibrated with ultrapure water. A 2-μL sample was pipetted and measured twice. The results showed a concentration of 51.8 ng/μL, an A260/280 ratio of 1.87, and an A260/230 ratio of 0.87. The calculated copy number was 7.24 × 10^6^ copies/μL. A 10-fold serial dilution was performed to obtain DNA templates ranging from 10^6^ to 10^0^ copies/μL. Comparative experiments were performed using LAMP, qPCR, and lateral flow assay (LFA) strips. The limit of detection (LOD) was assessed based on LAMP and qPCR fluorescence amplification curves, and standard curves were further generated by curve fitting. The PCR reaction conditions were 95 °C pre-denaturation for 1 min per cycle; 40 cycles of 95 °C denaturation for 10 s; and 50 °C annealing and extension for 30 s, with a ramp rate of 4 °C/s. The total reaction time was approximately 1 h and 8 min. The LAMP reaction conditions were 64 °C isothermal amplification for 60 s for 60 cycles, for a total reaction time of approximately 1 h and 7 min.

### 2.7. Specificity Assays

Genomic DNA from PA, *Mycobacterium tuberculosis*, *Helicobacter pylori*, *Escherichia coli*, and *Staphylococcus aureus* were subjected to LAMP to evaluate assay specificity. The presence or absence of specific amplification was assessed by fluorescence amplification curves and agarose gel electrophoresis. Each experiment was performed in triplicate.

### 2.8. Repeatability Experiments

Using the established LAMP assay for PA, DNA samples at concentrations of 10^6^ and 10^2^ copies/μL were selected for repeatability testing. Each sample was tested at least five times, and amplification was monitored via fluorescence curves. The normality of the fluorescence signal onset was evaluated, and the results were recorded for analysis. PA is widely present in nature, particularly in water, soil, and human skin. To adapt the system for on-site detection, we collected flowerbed soil and pond water near the hospital, boiled and lysed them, and purified them with the kit to verify its environmental adaptability.

### 2.9. Actual Samples

A total of 60 strains was obtained from Zhuzhou People’s Hospital. The strain was modeled as ATCC27853 and was isolated from human blood, and 2 μL of each template was added to the LAMP reaction system. Amplification was performed at 64 °C for 30 min, and fluorescence curves were monitored to evaluate successful signal initiation. The test results were subsequently recorded and analyzed.

## 3. Results and Discussion

In this study, we developed a novel LAMP-based detection system for PA, integrating both fluorescence and lateral flow strip assays to enable dual-mode detection. Compared with conventional methods, the proposed system significantly reduces the detection time to less than 20 min while maintaining high accuracy, enabling rapid and real-time on-site identification of PA. Owing to its portability and efficiency, this method is suitable for pathogen surveillance across various settings, including environmental, water, food, and clinical samples, thus providing a new technical pathway to address the globally recognized challenge of rapid on-site pathogen detection.

To ensure target specificity, the ecfX gene—known as a conserved and PA-specific genetic marker—was selected as the amplification target. A novel set of LAMP primers targeting the ecfX gene was successfully designed, screened, and validated, demonstrating excellent amplification efficiency and specificity.

Furthermore, the LAMP reaction parameters, including primer concentration, reaction temperature, and reaction time, were systematically optimized to enhance detection performance. The optimized system exhibited superior sensitivity, specificity, and stability. Validation using standard strains, water, and soil samples confirmed the robustness and reproducibility of the method, showing complete agreement with PCR-based detection results. These findings suggest that the optimized LAMP system provides a reliable and efficient platform for PA detection and may serve as a valuable methodological reference for extending LAMP-based assays to other pathogenic microorganisms.

### 3.1. Primer Screening

**Figure 1:**
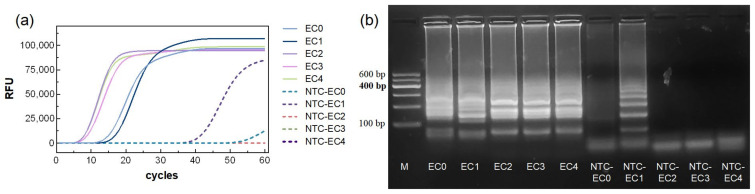
Primer screening for *Pseudomonas aeruginosa*. (**a**) qLAMP curves and (**b**) agarose gel electrophoresis of the amplification products.

The amplification curve results (Figure 1a) showed that all five primer sets (marked as EC0–EC4) produced increasing fluorescence signals in the reaction groups, exhibiting characteristic S-shaped curves, indicating successful amplification. The TTR for primers EC0 and EC1 was approximately 15 min, while primers EC2 and EC3 reached the TTR more rapidly, within 10 min. Among them, EC2 exhibited the fastest TTR, at around 8 min.

In the NTC groups, NTC-EC1 and NTC-EC0 showed fluorescence increases at approximately 40 min and 55 min, respectively, whereas NTC-EC2–EC4 showed no significant change, indicating no amplification. Agarose gel electrophoresis of the reaction products was consistent with the fluorescence results (Figure 1a). Reaction groups EC0–EC4 produced ladder-like bands, with bands from EC2–EC4 being brighter than those from EC0–EC1. In the NTC groups, NTC-EC1 exhibited non-specific bands, and NTC-EC0 showed faint bands, while NTC-EC2–EC4 displayed no bands. These results indicate that NTC-EC1 and NTC-EC0 may generate false positives and are therefore unsuitable primers. Among EC2–EC4, EC2 demonstrated the fastest TTR and the brightest agarose gel bands, indicating the best overall performance. Therefore, EC2 was selected as the optimal primer set for system optimization (Table 1).

### 3.2. Optimization of the Reaction System

**Figure 2:**
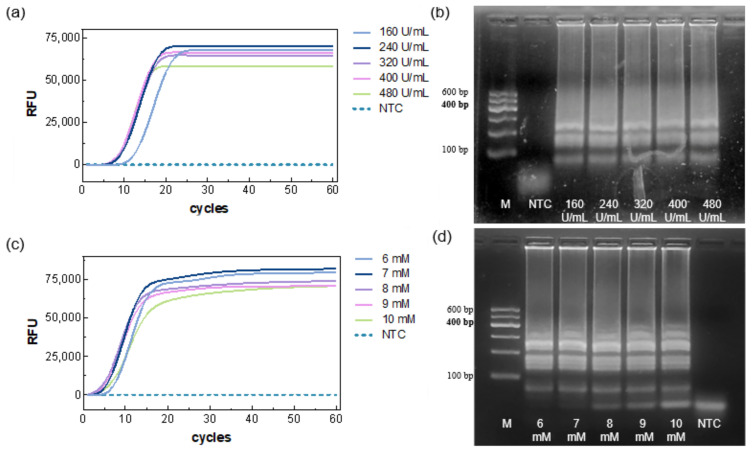
Optimization of enzyme concentration and Mg^2+^ concentration. Real-time LAMP (**a**,**b**) and agarose gel electrophoresis (**c**,**d**).

Using the selected primer marked EC2, the Bst polymerase concentration was first optimized. The results showed that Ct decreased as the enzyme concentration increased (Figure 2a), indicating faster amplification at higher enzyme levels. Specifically, the 160 U/mL group exhibited a slower onset (~13 min), whereas the 240–480 U/mL groups showed similar TTRs (~9.8–8.7 min); no amplification was observed in the NTC. Agarose gel electrophoresis results were consistent with the fluorescence data (Figure 2b), showing amplification bands for all five concentrations and no bands for the NTC. Considering reaction time and reagent economy, 320 U/mL was selected for subsequent experiments.

Based on the above optimized system, five Mg^2+^ concentrations were evaluated. All reaction groups generated amplification curves (Figure 2c), with the fastest TTR (~5.2 min) being observed at 8 mM. NTC remained negative. Gel electrophoresis showed amplification bands for all reactions, but primer–dimer bands appeared at the NTC position, with increasing brightness as Mg^2+^ concentration increased (Figure 2d), suggesting that excessive Mg^2+^ may neutralize the DNA backbone charge, reducing primer specificity. Both low and high Mg^2+^ adversely affected amplification efficiency. Therefore, 8 mM (including 2 mM from the buffer) was chosen as the optimal concentration.

**Figure 3:**
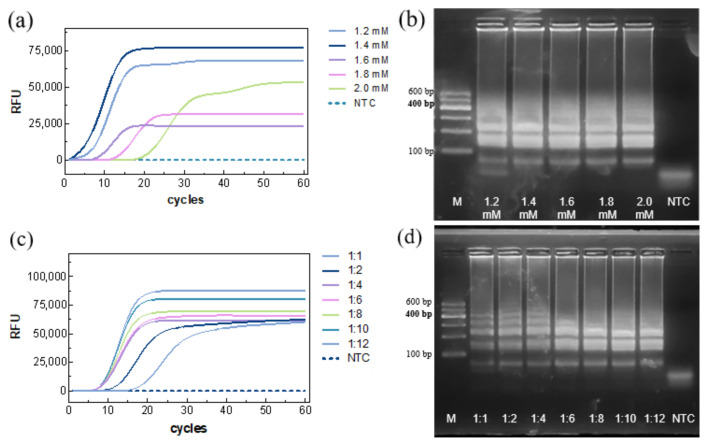
Optimization of dNTP concentration and primer ratio. (**a**,**b**) Real-time LAMP and (**c**,**d**) agarose gel electrophoresis.

The dNTP concentration was then optimized. Amplification curves indicated successful reactions for all groups (Figure 3a), with the fastest Ct (~5.91 min) at 1.4 mM. NTC remained negative. Gel electrophoresis showed that low dNTP concentrations resulted in incomplete amplification, whereas high concentrations complexed with Mg^2+^, inhibiting enzyme activity and slowing the reaction (Figure 3b). Thus, 1.4 mM was selected as the optimal dNTP concentration.

The inner/outer primer ratio was optimized next. Fluorescence detection showed the fastest TTR(~8.69 min) at a 1:8 ratio (Figure 3c), with no amplification in the NTC. Gel electrophoresis revealed bands for all seven tested ratios, with band intensity increasing as the ratio increased, and stabilizing at 1:8 (Figure 3d). Accordingly, an inner/outer primer ratio of 1:8 was chosen.

**Figure 4:**
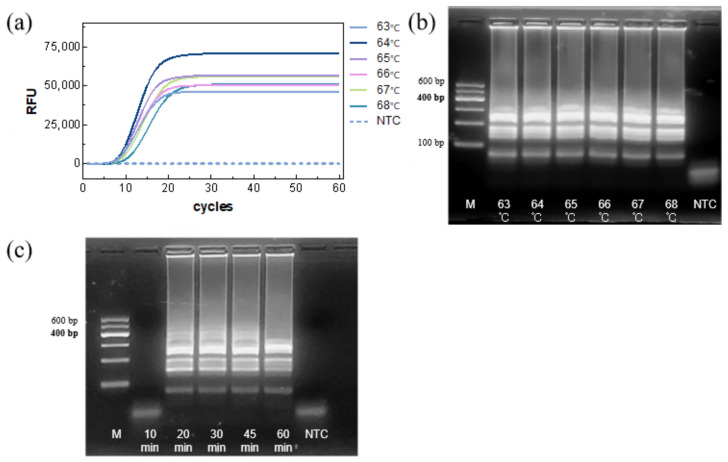
Optimization of reaction temperature and reaction time. (**a**) Real-time LAMP and (**b**,**c**) agarose gel electrophoresis.

The reaction temperature was then assessed. TTRs were similar across the tested temperatures, with 64 °C providing the highest amplification efficiency and fluorescence intensity (Figure 4a). Gel electrophoresis showed comparable band intensity across temperatures (Figure 4b), confirming 64 °C as the optimal temperature.

Finally, the reaction time was evaluated. Agarose gel electrophoresis showed clear amplification bands within 20 min, with little difference in band intensity at longer times (Figure 4c), indicating that complete amplification could be achieved within 20 min.

Finally, the optimal LAMP system for detection of the ecfX gene as show in Table 2.

### 3.3. Sensitivity of the LAMP Reaction System

**Figure 5:**
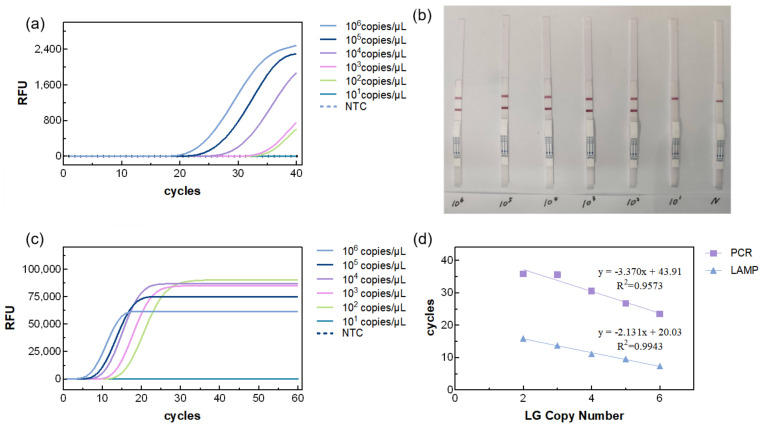
Sensitivity analysis of PA detection. (**a**) Amplification curves for real-time LAMP, (**b**) results for lateral flow assay, (**c**) amplification curves for real-time PCR, and (**d**) linear regression curves for qLAMP and qPCR.

LAMP, LFA, and qPCR assay were performed on DNA templates at different concentrations. The results showed that all three methods had the same limit of detection (LOD, 7.24 × 10^2^ copies/μL) (Figure 5a–c). However, for quantitative detection, the LAMP reaction exhibited a much shorter detection time (TTR: 7.33–15.9 min) compared with PCR (Ct: 23.45–35.86), and the correlation coefficient of LAMP (R^2^ = 0.9943) was higher than that of PCR (R^2^ = 0.9573) (Figure 5d). The minimum detection limits of the three methods are comparable. At the same concentration, the LAMP reaction yields results before the PCR reaction, and its amplification efficiency is higher than that of the PCR reaction. In addition, combining LAMP with LFA enables rapid qualitative analysis (Figure 5b).

### 3.4. Specificity of the LAMP Reaction System

**Figure 6:**
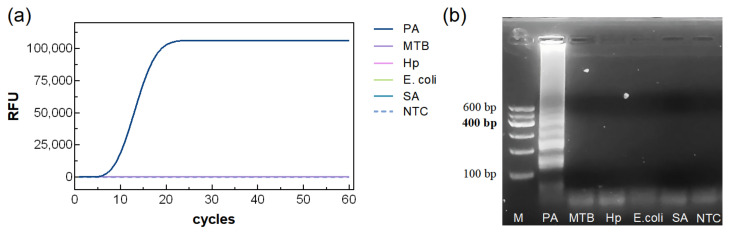
Specificity analysis of PA. (**a**) Amplification curves for real-time LAMP, (**b**) agarose gel electrophoresis.

Using the optimized LAMP system, DNA templates from PA and four other bacterial species were tested. As shown in Figure 6, only PA produced a clear amplification curve and visible bands on agarose gel, demonstrating the high specificity of the assay.

### 3.5. Repeatability of the LAMP Reaction System

**Figure 7:**
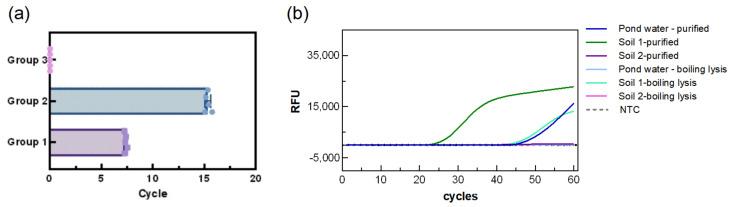
Repeatability of the LAMP reaction system. (**a**) Statistical analysis of TTRs for different PA sample groups. Group 1 and Group 2 were experimental groups and Group 3 was the control group, (**b**) impact of water and soil environment on detection,.

Based on different DNA concentrations, samples were divided into two experimental groups and one control group, with each group tested in five replicates. As shown in Figure 7, the mean fluorescence amplification time of the first group was (7.3 ± 0.1 min, CV:1.568%), while the second group showed a mean time of (15.3 ± 0.2, CV:1.827%) min. No amplification was observed in the control group. The low standard deviations in both experimental groups indicate that the assay is highly reproducible and stable. Regarding adaptability to the environment, the results showed that the system was able to detect the soil and water samples purified with the kit, albeit with relatively long detection times (TTR: 26.34 min for soil 1, 35.89 min for soil 2, and 48.97 min for pond water). Surprisingly, the system was able to directly detect PA in soil 1 (TTR: 46.21 min), but not in pond water and soil 2, suggesting a certain environmental influence which is possibly related to the low bacterial count in the water samples.

### 3.6. Detection of Actual Samples

**Figure 8:**
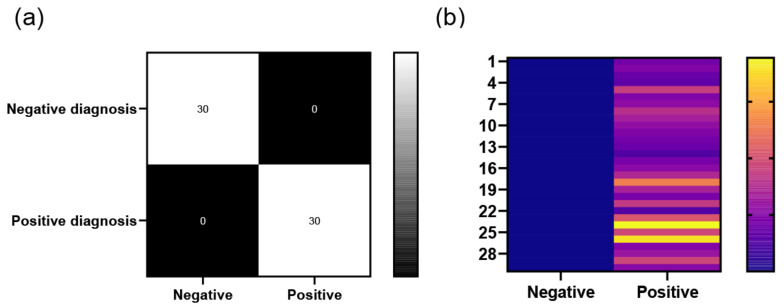
(**a**) The number of positive, positive test, negative, negative test and (**b**) heatmap of TTRs for 60 clinical samples.

The LAMP reaction was performed on 60 standard strain samples. The sensitivity was 100% (CL: 90.5%, 100%), specificity was 100% (CL: 90.5%, 100%), positive predictive value was 100% (CL: 90.5%, 100%), and the negative predictive value was 100% (CL: 90.5%, 100%). The fluorescence amplification start times were visualized as a heatmap. As shown in Figure 8, negative samples exhibited no significant changes in fluorescence intensity, whereas all positive samples showed clear amplification. These results demonstrate that the assay is reliable.

## 4. Conclusions

In this study, ten LAMP primer sets targeting the PA ecfX gene were designed and screened, resulting in the selection of the optimal primer set, EC2. Using EC2, the reaction conditions and components were systematically optimized. The assay performance was further validated by the comparison with LFA strips and conventional PCR. The optimized LAMP system for PA was established as follows: Bst enzyme concentration 320 U/mL, Mg^2+^ 8 mM, dNTPs 1.4 mM, inner-to-outer primer ratio 1:8, and reaction at 64 °C for a minimum of 20 min. The detection limit of this system was comparable to that of PCR and LFA strips, with a significantly shorter reaction time, and demonstrated high specificity and reproducibility. As a highly specific, rapid, and stable method for PA detection, it is widely applied for the rapid screening of clinical, water, and environmental samples. Moreover, the established ecfX-LAMP assay provides a reliable methodological framework that can be adapted for the detection of other pathogens across diverse settings.

**Limitations:** This study validated the specificity of the assay against only four nontargeted strains. To further enhance the robustness and clinical applicability of this method, subsequent studies should expand the validation scope to include more common clinically available Gram-negative bacteria with similar pathogenic characteristics, such as Klebsiella pneumoniae and Acinetobacter baumannii. Secondly, the research on environmental samples does not go into enough depth. More water and soil samples are needed for verification in the future. A LAMP method for PA detection demonstrated good sensitivity and specificity, as well as a relatively short detection time. If combined with other approaches (such as CRISPR-based detection), its signal output could be further enhanced.

## Figures and Tables

**Table 1:** Primer sequences for the ecfX gene used for LAMP.

Primer Name	Primer Sequence	%GC	Tm	Primer Length
F3	AAGTTGCGGGCGATCTG	58.82	56.10	17
B3	TCCGTGGTTCCGTCTCG	64.71	57.82	17
FIP	GACCTCGCCCAGGATACTTTCGGGCTGCTCGACCGATTG	61.54	73.76	39
BIP	CCGAACTGCCCAGGTGCTTGCCTATCAGGCGTTCCATG	60.53	73.55	38
LF	CCCAGTGGCTGAAATGGC	61.11	57.49	18
LB	CGCAGGAAGCGCAGCAA	64.71	60.35	17

**Table 2:** The optimal LAMP system is as follows.

Composition		Volume	Final Concentration
10× IsothermalAmp Buffer		2.5 μL	1×
MgSO_4_ (100 mM)		1.5 μL	6 mM (total 8 mM)
dNTP Mix (10 mM each)		3.5 μL	1.4 mM
Primer pre-mix	FIP/BIP Primers (16×)	2.5 μL	1.6 μM
	F3/B3 Primers (2×)		0.2 μM
	LoopF/B Primers (4×)		0.4 μM
Bst II Pro DNA Polymerase Large Fragment (8 U/μL)		1.0 μL	0.32 U/μL
50× LAMP Fluorescent Dye		0.5 μL	1×
DNA Template		2.0 μL	/
Nuclease-free ddH_2_O		Up to 25 μL	/

## Data Availability

The original contributions presented in this study are included in the article. Further inquiries can be directed to the corresponding author.

## Supplementary Materials

The following supporting information can be downloaded at: https://www.mdpi.com/article/10.3390/bios15110750/s1.

## Abbreviations

The following abbreviations are used in this manuscript:

PA	*Pseudomonas aeruginosa*
NTC	No-template control
LAMP	Loop-mediated isothermal amplification
qPCR	Real-time polymerase chain reaction
qLAMP	Real-time loop-mediated isothermal amplification
TTR	Time to results
LFA	Lateral flow assay
LOD	Limit of detection
