# Supplementary material for: Rapid On-Site Detection of Pseudomonas aeruginosa via ecfX-Targeted Loop-Mediated Isothermal Amplification
Source: Biosensors (Basel). 2025 Nov 7;15(11):750. doi: 10.3390/bios15110750 (PMC12649875; doi:10.3390/bios15110750)
Supplement: Supplementary file 1 [file biosensors-15-00750-s001.zip › biosensors-3903822-supplementary.pdf]

The homology of the primers of EC2 gene with the NCBI genome was analyzed using BLAST, and the results are as follows:

F3：AAGTTGCGGGCGATCTG

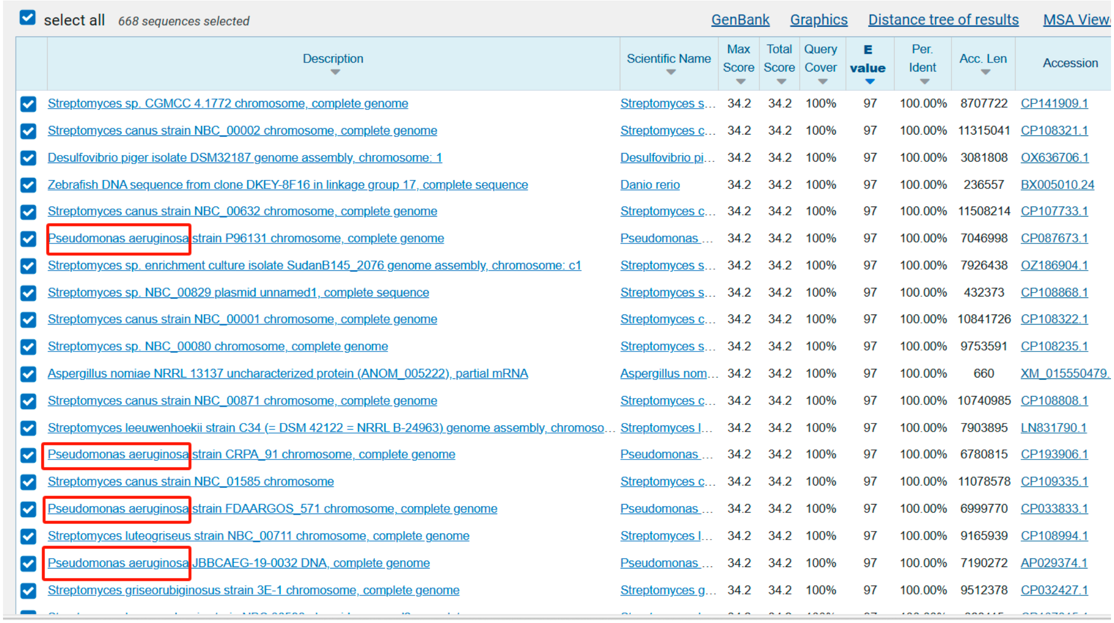

select all 668 sequences selected — GenBank Graphics Distance tree of results MSA View

Description	Scientific Name	Max Score	Total Score	Query Cover	E value	Per. Ident	Acc. Len	Accession
Streptomyces sp. CGMCC 4.1772 chromosome, complete genome	Streptomyces s...	34.2	34.2	100%	97	100.00%	8707722	CP141909.1
Streptomyces canus strain NBC_00002 chromosome, complete genome	Streptomyces c...	34.2	34.2	100%	97	100.00%	11315041	CP108321.1
Desulfovibrio piger isolate DSM32187 genome assembly, chromosome: 1	Desulfovibrio pi...	34.2	34.2	100%	97	100.00%	3081808	OX636706.1
Zebrafish DNA sequence from clone DKEY-8F16 in linkage group 17, complete sequence	Danio rerio	34.2	34.2	100%	97	100.00%	236557	BX005010.24
Streptomyces canus strain NBC_00632 chromosome, complete genome	Streptomyces c...	34.2	34.2	100%	97	100.00%	11508214	CP107733.1
Pseudomonas aeruginosa strain P96131 chromosome, complete genome	Pseudomonas ...	34.2	34.2	100%	97	100.00%	7046998	CP087673.1
Streptomyces sp. enrichment culture isolate SudanB145_2076 genome assembly, chromosome: c1	Streptomyces s...	34.2	34.2	100%	97	100.00%	7926438	OZ186904.1
Streptomyces sp. NBC_00829 plasmid unnamed1, complete sequence	Streptomyces s...	34.2	34.2	100%	97	100.00%	432373	CP108868.1
Streptomyces canus strain NBC_00001 chromosome, complete genome	Streptomyces c...	34.2	34.2	100%	97	100.00%	10841726	CP108322.1
Streptomyces sp. NBC_00080 chromosome, complete genome	Streptomyces s...	34.2	34.2	100%	97	100.00%	9753591	CP108235.1
Aspergillus nomiae NRRL 13137 uncharacterized protein (ANOM_005222), partial mRNA	Aspergillus nom...	34.2	34.2	100%	97	100.00%	660	XM_015550479.
Streptomyces canus strain NBC_00871 chromosome, complete genome	Streptomyces c...	34.2	34.2	100%	97	100.00%	10740985	CP108808.1
Streptomyces leeuwenhoekii strain C34 (= DSM 42122 = NRRL B-24963) genome assembly, chromoso...	Streptomyces l...	34.2	34.2	100%	97	100.00%	7903895	LN831790.1
Pseudomonas aeruginosa strain CRPA_91 chromosome, complete genome	Pseudomonas ...	34.2	34.2	100%	97	100.00%	6780815	CP193906.1
Streptomyces canus strain NBC_01585 chromosome	Streptomyces c...	34.2	34.2	100%	97	100.00%	11078578	CP109335.1
Pseudomonas aeruginosa strain FDAARGOS_571 chromosome, complete genome	Pseudomonas ...	34.2	34.2	100%	97	100.00%	6999770	CP033833.1
Streptomyces luteogriseus strain NBC_00711 chromosome, complete genome	Streptomyces l...	34.2	34.2	100%	97	100.00%	9165939	CP108994.1
Pseudomonas aeruginosa JBBCAEG-19-0032 DNA, complete genome	Pseudomonas ...	34.2	34.2	100%	97	100.00%	7190272	AP029374.1
Streptomyces griseorubiginosus strain 3E-1 chromosome, complete genome	Streptomyces g...	34.2	34.2	100%	97	100.00%	9512378	CP032427.1

Alignment results showed that the F3 primer exhibited homology with other species in addition to Pseudomonas aeruginosa when compared against the entire NCBI database.

B3：TCCGTGGTTCCGTCTCG

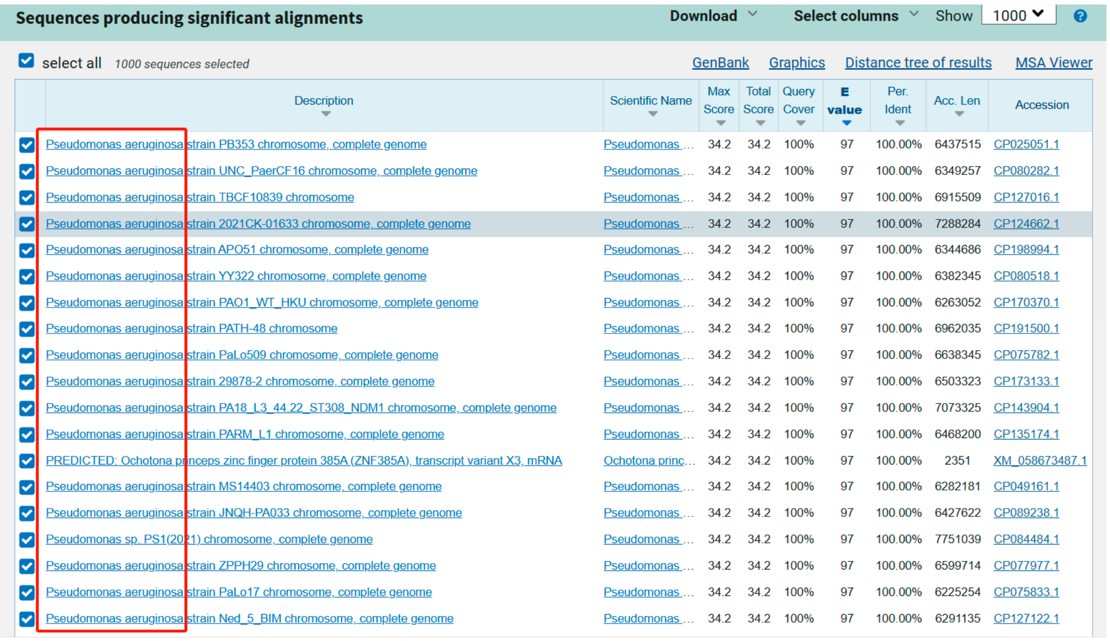

Sequences producing significant alignments — Download Select columns Show 1000

select all 1000 sequences selected — GenBank Graphics Distance tree of results MSA Viewer

Description	Scientific Name	Max Score	Total Score	Query Cover	E value	Per. Ident	Acc. Len	Accession
Pseudomonas aeruginosa strain PB353 chromosome, complete genome	Pseudomonas ...	34.2	34.2	100%	97	100.00%	6437515	CP025051.1
Pseudomonas aeruginosa strain UNC_PaerCF16 chromosome, complete genome	Pseudomonas ...	34.2	34.2	100%	97	100.00%	6349257	CP080282.1
Pseudomonas aeruginosa strain TBCF10839 chromosome	Pseudomonas ...	34.2	34.2	100%	97	100.00%	6915509	CP127016.1
Pseudomonas aeruginosa strain 2021CK-01633 chromosome, complete genome	Pseudomonas ...	34.2	34.2	100%	97	100.00%	7288284	CP124662.1
Pseudomonas aeruginosa strain APO51 chromosome, complete genome	Pseudomonas ...	34.2	34.2	100%	97	100.00%	6344686	CP198994.1
Pseudomonas aeruginosa strain YY322 chromosome, complete genome	Pseudomonas ...	34.2	34.2	100%	97	100.00%	6382345	CP080518.1
Pseudomonas aeruginosa strain PAO1_WT_HKU chromosome, complete genome	Pseudomonas ...	34.2	34.2	100%	97	100.00%	6263052	CP170370.1
Pseudomonas aeruginosa strain PATH-48 chromosome	Pseudomonas ...	34.2	34.2	100%	97	100.00%	6962035	CP191500.1
Pseudomonas aeruginosa strain PaLo509 chromosome, complete genome	Pseudomonas ...	34.2	34.2	100%	97	100.00%	6638345	CP075782.1
Pseudomonas aeruginosa strain 29878-2 chromosome, complete genome	Pseudomonas ...	34.2	34.2	100%	97	100.00%	6503323	CP173133.1
Pseudomonas aeruginosa strain PA18_L3_44.22_ST308_NDM1 chromosome, complete genome	Pseudomonas ...	34.2	34.2	100%	97	100.00%	7073325	CP143904.1
Pseudomonas aeruginosa strain PARM_L1 chromosome, complete genome	Pseudomonas ...	34.2	34.2	100%	97	100.00%	6468200	CP135174.1
PREDICTED: Ochotona princeps zinc finger protein 385A (ZNF385A), transcript variant X3, mRNA	Ochotona princ...	34.2	34.2	100%	97	100.00%	2351	XM_058673487.1
Pseudomonas aeruginosa strain MS14403 chromosome, complete genome	Pseudomonas ...	34.2	34.2	100%	97	100.00%	6282181	CP049161.1
Pseudomonas aeruginosa strain JNQH-PA033 chromosome, complete genome	Pseudomonas ...	34.2	34.2	100%	97	100.00%	6427622	CP089238.1
Pseudomonas sp. PS1(20[illegible]1) chromosome, complete genome	Pseudomonas ...	34.2	34.2	100%	97	100.00%	7751039	CP084484.1
Pseudomonas aeruginosa strain ZPPH29 chromosome, complete genome	Pseudomonas ...	34.2	34.2	100%	97	100.00%	6599714	CP077977.1
Pseudomonas aeruginosa strain PaLo17 chromosome, complete genome	Pseudomonas ...	34.2	34.2	100%	97	100.00%	6225254	CP075833.1
Pseudomonas aeruginosa strain Ned_5_BIM chromosome, complete genome	Pseudomonas ...	34.2	34.2	100%	97	100.00%	6291135	CP127122.1

Alignment results showed that the B3 primer exhibited homology exclusively with Pseudomonas aeruginosa when compared against the entire NCBI database.

FIP：GACCTCGCCCAGGATACTTTCG-GGCTGCTCGACCGATTG

select all 1000 sequences selected | GenBank | Graphics | Distance tree of results | MSA Viewer

Description	Scientific Name	Max Score	Total Score	Query Cover	E value	Per. Ident	Acc. Len	Accession
Pseudomonas aeruginosa strain PB353 chromosome, complete genome	Pseudomonas aeruginosa	44.1	44.1	100%	0.10	100.00%	6437515	CP025051.1
Pseudomonas aeruginosa strain UNC_PaerCF16 chromosome, complete genome	Pseudomonas aeruginosa	44.1	44.1	100%	0.10	100.00%	6349257	CP080282.1
Pseudomonas aeruginosa strain TBCF10839 chromosome	Pseudomonas aeruginosa	44.1	44.1	100%	0.10	100.00%	6915509	CP127016.1
Pseudomonas aeruginosa strain 2021CK-01633 chromosome, complete genome	Pseudomonas aeruginosa	44.1	44.1	100%	0.10	100.00%	7288284	CP124662.1
Pseudomonas aeruginosa strain APO51 chromosome, complete genome	Pseudomonas aeruginosa	44.1	44.1	100%	0.10	100.00%	6344686	CP198994.1
Pseudomonas aeruginosa strain YY322 chromosome, complete genome	Pseudomonas aeruginosa	44.1	44.1	100%	0.10	100.00%	6382345	CP080518.1
Pseudomonas aeruginosa strain PAO1_WT_HKU chromosome, complete genome	Pseudomonas aeruginosa	44.1	44.1	100%	0.10	100.00%	6263052	CP170370.1
Pseudomonas aeruginosa strain PATH-48 chromosome	Pseudomonas aeruginosa	44.1	44.1	100%	0.10	100.00%	6962035	CP191500.1
Pseudomonas aeruginosa strain PaLo509 chromosome, complete genome	Pseudomonas aeruginosa	44.1	44.1	100%	0.10	100.00%	6638345	CP075782.1
Pseudomonas aeruginosa strain 29878-2 chromosome, complete genome	Pseudomonas aeruginosa	44.1	44.1	100%	0.10	100.00%	6503323	CP173133.1
Pseudomonas aeruginosa strain PA18_L3_44.22_ST308_NDM1 chromosome, complet…	Pseudomonas aeruginosa	44.1	44.1	100%	0.10	100.00%	7073325	CP143904.1
Pseudomonas aeruginosa strain PARM_L1 chromosome, complete genome	Pseudomonas aeruginosa	44.1	44.1	100%	0.10	100.00%	6468200	CP135174.1
Pseudomonas aeruginosa strain MS14403 chromosome, complete genome	Pseudomonas aeruginosa	44.1	44.1	100%	0.10	100.00%	6282181	CP049161.1
Pseudomonas aeruginosa strain JNQH-PA033 chromosome, complete genome	Pseudomonas aeruginosa	44.1	44.1	100%	0.10	100.00%	6427622	CP089238.1
Pseudomonas sp. PS1(2021) chromosome, complete genome	Pseudomonas sp. PS1(2021)	44.1	44.1	100%	0.10	100.00%	7751039	CP084484.1
Pseudomonas aeruginosa strain ZPPH29 chromosome, complete genome	Pseudomonas aeruginosa	44.1	44.1	100%	0.10	100.00%	6599714	CP077977.1
Pseudomonas aeruginosa strain PaLo17 chromosome, complete genome	Pseudomonas aeruginosa	44.1	44.1	100%	0.10	100.00%	6225254	CP075833.1
Pseudomonas aeruginosa strain Ned_5_BIM chromosome, complete genome	Pseudomonas aeruginosa	44.1	44.1	100%	0.10	100.00%	6291135	CP127122.1
Pseudomonas aeruginosa strain Pa021 chromosome, complete genome	Pseudomonas aeruginosa	44.1	44.1	100%	0.10	100.00%	6334617	CP177307.1

Alignment results showed that the FIP primer region 1 exhibited homology exclusively with Pseudomonas aeruginosa when compared against the entire NCBI database.

select all 1000 sequences selected | GenBank | Graphics | Distance tree of results | MSA Viewer

Description	Scientific Name	Max Score	Total Score	Query Cover	E value	Per. Ident	Acc. Len	Accession
Micromonospora zamorensis strain NBC_00407 chromosome, complete genome	Micromonospor…	34.2	34.2	100%	97	100.00%	7313367	CP107936.1
Pseudomonas aeruginosa strain PB353 chromosome, complete genome	Pseudomonas …	34.2	34.2	100%	97	100.00%	6437515	CP025051.1
Pseudomonas aeruginosa strain UNC_PaerCF16 chromosome, complete genome	Pseudomonas …	34.2	34.2	100%	97	100.00%	6349257	CP080282.1
Pseudomonas aeruginosa strain TBCF10839 chromosome	Pseudomonas …	34.2	34.2	100%	97	100.00%	6915509	CP127016.1
Pseudomonas aeruginosa strain 2021CK-01633 chromosome, complete genome	Pseudomonas …	34.2	34.2	100%	97	100.00%	7288284	CP124662.1
Pseudomonas aeruginosa strain APO51 chromosome, complete genome	Pseudomonas …	34.2	34.2	100%	97	100.00%	6344686	CP198994.1
Pseudomonas aeruginosa strain YY322 chromosome, complete genome	Pseudomonas …	34.2	34.2	100%	97	100.00%	6382345	CP080518.1
Pseudomonas aeruginosa strain PAO1_WT_HKU chromosome, complete genome	Pseudomonas …	34.2	34.2	100%	97	100.00%	6263052	CP170370.1
Pseudomonas aeruginosa strain PATH-48 chromosome	Pseudomonas …	34.2	34.2	100%	97	100.00%	6962035	CP191500.1
Pseudomonas aeruginosa strain PaLo509 chromosome, complete genome	Pseudomonas …	34.2	34.2	100%	97	100.00%	6638345	CP075782.1
Pseudomonas aeruginosa strain 29878-2 chromosome, complete genome	Pseudomonas …	34.2	34.2	100%	97	100.00%	6503323	CP173133.1
Pseudomonas aeruginosa strain PA18_L3_44.22_ST308_NDM1 chromosome, complete genome	Pseudomonas …	34.2	34.2	100%	97	100.00%	7073325	CP143904.1
Pseudomonas aeruginosa strain PARM_L1 chromosome, complete genome	Pseudomonas …	34.2	34.2	100%	97	100.00%	6468200	CP135174.1
Pseudomonas aeruginosa strain MS14403 chromosome, complete genome	Pseudomonas …	34.2	34.2	100%	97	100.00%	6282181	CP049161.1
Pseudomonas sp. PS1(2021) chromosome, complete genome	Pseudomonas …	34.2	34.2	100%	97	100.00%	7751039	CP084484.1
Pseudomonas aeruginosa strain ZPPH29 chromosome, complete genome	Pseudomonas …	34.2	34.2	100%	97	100.00%	6599714	CP077977.1
Pseudomonas aeruginosa strain PaLo17 chromosome, complete genome	Pseudomonas …	34.2	34.2	100%	97	100.00%	6225254	CP075833.1
Pseudomonas aeruginosa strain Ned_5_BIM chromosome, complete genome	Pseudomonas …	34.2	34.2	100%	97	100.00%	6291135	CP127122.1
Pseudomonas aeruginosa strain Pa021 chromosome, complete genome	Pseudomonas …	34.2	34.2	100%	97	100.00%	6334617	CP177307.1

Alignment results showed that over 99% of BLAST hits for FIP primer region 2 exhibited homology exclusively with Pseudomonas aeruginosa when compared against the entire NCBI database.

BIP：CCGAACTGCCCAGGTGCTTGC-CTATCAGGCGTTCCATG

select all *1000 sequences selected* GenBank Graphics Distance tree of results MSA Viewer

Description	Scientific Name	Max Score	Total Score	Query Cover	E value	Per. Ident	Acc. Len	Accession
Pseudomonas aeruginosa strain PB353 chromosome, complete genome	Pseudomonas aeruginosa	42.1	42.1	100%	0.40	100.00%	6437515	CP025051.1
Pseudomonas aeruginosa strain UNC_PaerCF16 chromosome, complete genome	Pseudomonas aeruginosa	42.1	42.1	100%	0.40	100.00%	6349257	CP080282.1
Pseudomonas aeruginosa strain 2021CK-01633 chromosome, complete genome	Pseudomonas aeruginosa	42.1	42.1	100%	0.40	100.00%	7288284	CP124662.1
Pseudomonas aeruginosa strain APO51 chromosome, complete genome	Pseudomonas aeruginosa	42.1	42.1	100%	0.40	100.00%	6344686	CP198994.1
Pseudomonas aeruginosa strain YY322 chromosome, complete genome	Pseudomonas aeruginosa	42.1	42.1	100%	0.40	100.00%	6382345	CP080518.1
Pseudomonas aeruginosa strain PATH-48 chromosome	Pseudomonas aeruginosa	42.1	42.1	100%	0.40	100.00%	6962035	CP191500.1
Pseudomonas aeruginosa strain PaLo509 chromosome, complete genome	Pseudomonas aeruginosa	42.1	42.1	100%	0.40	100.00%	6638345	CP075782.1
Pseudomonas aeruginosa strain 29878-2 chromosome, complete genome	Pseudomonas aeruginosa	42.1	42.1	100%	0.40	100.00%	6503323	CP173133.1
Pseudomonas aeruginosa strain PA18_L3_44.22_ST308_NDM1 chromosome, complete genome	Pseudomonas aeruginosa	42.1	42.1	100%	0.40	100.00%	7073325	CP143904.1
Pseudomonas aeruginosa strain PARM_L1 chromosome, complete genome	Pseudomonas aeruginosa	42.1	42.1	100%	0.40	100.00%	6468200	CP135174.1
Pseudomonas aeruginosa strain MS14403 chromosome, complete genome	Pseudomonas aeruginosa	42.1	42.1	100%	0.40	100.00%	6282181	CP049161.1
Pseudomonas aeruginosa strain JNQH-PA033 chromosome, complete genome	Pseudomonas aeruginosa	42.1	42.1	100%	0.40	100.00%	6427622	CP089238.1
Pseudomonas sp. PS1(2021) chromosome, complete genome	Pseudomonas sp. PS1(2021)	42.1	42.1	100%	0.40	100.00%	7751039	CP084484.1
Pseudomonas aeruginosa strain ZPPH29 chromosome, complete genome	Pseudomonas aeruginosa	42.1	42.1	100%	0.40	100.00%	6599714	CP077977.1
Pseudomonas aeruginosa strain PaLo17 chromosome, complete genome	Pseudomonas aeruginosa	42.1	42.1	100%	0.40	100.00%	6225254	CP075833.1
Pseudomonas aeruginosa strain Pa021 chromosome, complete genome	Pseudomonas aeruginosa	42.1	42.1	100%	0.40	100.00%	6334617	CP177307.1
Pseudomonas aeruginosa strain strain KUD2 chromosome, complete genome	Pseudomonas aeruginosa	42.1	42.1	100%	0.40	100.00%	6941065	CP138577.1
Pseudomonas aeruginosa strain CF-sbj4-Pa23-I chromosome, complete genome	Pseudomonas aeruginosa	42.1	42.1	100%	0.40	100.00%	6271205	CP199827.1
Pseudomonas aeruginosa strain WTJH36 chromosome, complete genome	Pseudomonas aeruginosa	42.1	42.1	100%	0.40	100.00%	7094521	CP104590.1
Pseudomonas aeruginosa strain 2021CK-01445 chromosome, complete genome	Pseudomonas aeruginosa	42.1	42.1	100%	0.40	100.00%	6581794	CP124668.1

Alignment results showed that BIP primer region 1 exhibited homology exclusively with Pseudomonas aeruginosa when compared against the entire NCBI database.

select all *1000 sequences selected* GenBank Graphics Distance tree of results MSA Viev

Description	Scientific Name	Max Score	Total Score	Query Cover	E value	Per. Ident	Acc. Len	Accession
Pseudomonas aeruginosa strain PB353 chromosome, complete genome	Pseudomonas ...	34.2	34.2	100%	97	100.00%	6437515	CP025051.1
Pseudomonas aeruginosa strain UNC_PaerCF16 chromosome, complete genome	Pseudomonas ...	34.2	34.2	100%	97	100.00%	6349257	CP080282.1
Pseudomonas aeruginosa strain TBCF10839 chromosome	Pseudomonas ...	34.2	34.2	100%	97	100.00%	6915509	CP127016.1
Pseudomonas aeruginosa strain 2021CK-01633 chromosome, complete genome	Pseudomonas ...	34.2	34.2	100%	97	100.00%	7288284	CP124662.1
Pseudomonas aeruginosa strain APO51 chromosome, complete genome	Pseudomonas ...	34.2	34.2	100%	97	100.00%	6344686	CP198994.1
Pseudomonas aeruginosa strain YY322 chromosome, complete genome	Pseudomonas ...	34.2	34.2	100%	97	100.00%	6382345	CP080518.1
Pseudomonas aeruginosa strain PAO1_WT_HKU chromosome, complete genome	Pseudomonas ...	34.2	34.2	100%	97	100.00%	6263052	CP170370.1
Pseudomonas aeruginosa strain PATH-48 chromosome	Pseudomonas ...	34.2	34.2	100%	97	100.00%	6962035	CP191500.1
Pseudomonas aeruginosa strain PaLo509 chromosome, complete genome	Pseudomonas ...	34.2	34.2	100%	97	100.00%	6638345	CP075782.1
Pseudomonas aeruginosa strain 29878-2 chromosome, complete genome	Pseudomonas ...	34.2	34.2	100%	97	100.00%	6503323	CP173133.1
Pseudomonas aeruginosa strain PA18_L3_44.22_ST308_NDM1 chromosome, complete genome	Pseudomonas ...	34.2	34.2	100%	97	100.00%	7073325	CP143904.1
Pseudomonas aeruginosa strain PARM_L1 chromosome, complete genome	Pseudomonas ...	34.2	34.2	100%	97	100.00%	6468200	CP135174.1
Pseudomonas aeruginosa strain MS14403 chromosome, complete genome	Pseudomonas ...	34.2	34.2	100%	97	100.00%	6282181	CP049161.1
Pseudomonas aeruginosa strain JNQH-PA033 chromosome, complete genome	Pseudomonas ...	34.2	34.2	100%	97	100.00%	6427622	CP089238.1
Pseudomonas sp. PS1(2021) chromosome, complete genome	Pseudomonas ...	34.2	34.2	100%	97	100.00%	7751039	CP084484.1
Pseudomonas aeruginosa strain ZPPH29 chromosome, complete genome	Pseudomonas ...	34.2	34.2	100%	97	100.00%	6599714	CP077977.1
Pseudomonas aeruginosa strain PaLo17 chromosome, complete genome	Pseudomonas ...	34.2	34.2	100%	97	100.00%	6225254	CP075833.1
Pseudomonas aeruginosa strain Ned_5_BIM chromosome, complete genome	Pseudomonas ...	34.2	34.2	100%	97	100.00%	6291135	CP127122.1
Pseudomonas aeruginosa strain Pa021 chromosome, complete genome	Pseudomonas ...	34.2	34.2	100%	97	100.00%	6334617	CP177307.1
Pseudomonas aeruginosa PA99 chromosome, complete genome	Pseudomonas ...	34.2	34.2	100%	97	100.00%	6946480	CP042967.1

Alignment results showed that BIP primer region 2 exhibited homology exclusively with Pseudomonas aeruginosa when compared against the entire NCBI database.

LF：CCCAGTGGCTGAAATGGC

select all 1000 sequences selected GenBank Graphics Distance tree of results MSA View

Description	Scientific Name	Max Score	Total Score	Query Cover	E value	Per. Ident	Acc. Len	Accession
Pseudomonas aeruginosa strain PB353 chromosome, complete genome	Pseudomonas ...	36.2	36.2	100%	24	100.00%	6437515	CP025051.1
Pseudomonas aeruginosa strain UNC_PaerCF16 chromosome, complete genome	Pseudomonas ...	36.2	36.2	100%	24	100.00%	6349257	CP080282.1
Pseudomonas aeruginosa strain TBCF10839 chromosome	Pseudomonas ...	36.2	36.2	100%	24	100.00%	6915509	CP127016.1
Pseudomonas aeruginosa strain 2021CK-01633 chromosome, complete genome	Pseudomonas ...	36.2	36.2	100%	24	100.00%	7288284	CP124662.1
Pseudomonas aeruginosa strain APO51 chromosome, complete genome	Pseudomonas ...	36.2	36.2	100%	24	100.00%	6344686	CP198994.1
Pseudomonas aeruginosa strain YY322 chromosome, complete genome	Pseudomonas ...	36.2	36.2	100%	24	100.00%	6382345	CP080518.1
Pseudomonas aeruginosa strain PAO1_WT_HKU chromosome, complete genome	Pseudomonas ...	36.2	36.2	100%	24	100.00%	6263052	CP170370.1
Pseudomonas aeruginosa strain PATH-48 chromosome	Pseudomonas ...	36.2	36.2	100%	24	100.00%	6962035	CP191500.1
Pseudomonas aeruginosa strain PaLo509 chromosome, complete genome	Pseudomonas ...	36.2	36.2	100%	24	100.00%	6638345	CP075782.1
Pseudomonas aeruginosa strain 29878-2 chromosome, complete genome	Pseudomonas ...	36.2	36.2	100%	24	100.00%	6503323	CP173133.1
Pseudomonas aeruginosa strain PA18_L3_44.22_ST308_NDM1 chromosome, complete genome	Pseudomonas ...	36.2	36.2	100%	24	100.00%	7073325	CP143904.1
Pseudomonas aeruginosa strain PARM_L1 chromosome, complete genome	Pseudomonas ...	36.2	36.2	100%	24	100.00%	6468200	CP135174.1
Pseudomonas aeruginosa strain MS14403 chromosome, complete genome	Pseudomonas ...	36.2	36.2	100%	24	100.00%	6282181	CP049161.1
Pseudomonas aeruginosa strain JNQH-PA033 chromosome, complete genome	Pseudomonas ...	36.2	36.2	100%	24	100.00%	6427622	CP089238.1
Pseudomonas sp. PS1(20[illegible]1) chromosome, complete genome	Pseudomonas ...	36.2	36.2	100%	24	100.00%	7751039	CP084484.1
Pseudomonas aeruginosa strain ZPPH29 chromosome, complete genome	Pseudomonas ...	36.2	36.2	100%	24	100.00%	6599714	CP077977.1
Pseudomonas aeruginosa strain PaLo17 chromosome, complete genome	Pseudomonas ...	36.2	36.2	100%	24	100.00%	6225254	CP075833.1
Pseudomonas aeruginosa strain Ned_5_BIM chromosome, complete genome	Pseudomonas ...	36.2	36.2	100%	24	100.00%	6291135	CP127122.1
Pseudomonas aeruginosa strain Pa021 chromosome, complete genome	Pseudomonas ...	36.2	36.2	100%	24	100.00%	6334617	CP177307.1

Alignment results showed that the LF primer exhibited homology exclusively with Pseudomonas aeruginosa when compared against the entire NCBI database.

LB：CGCAGGAAGCGCAGCAA

select all 999 sequences selected GenBank Graphics Distance tree of results MSA Viewer

Description	Scientific Name	Max Score	Total Score	Query Cover	E value	Per. Ident	Acc. Len	Accession
Cupriavidus sp. P-10 DNA, chromosome 2, complete sequence	Cupriavidus sp....	34.2	34.2	100%	97	100.00%	2929165	AP025171.1
Pseudomonas aeruginosa strain MS14403 chromosome, complete genome	Pseudomonas ...	34.2	98.6	100%	97	100.00%	6282181	CP049161.1
Pseudomonas aeruginosa strain JNQH-PA033 chromosome, complete genome	Pseudomonas ...	34.2	98.6	100%	97	100.00%	6427622	CP089238.1
Pseudomonas sp. PS1(20[illegible]1) chromosome, complete genome	Pseudomonas ...	34.2	98.6	100%	97	100.00%	7751039	CP084484.1
Pseudomonas aeruginosa strain ZPPH29 chromosome, complete genome	Pseudomonas ...	34.2	98.6	100%	97	100.00%	6599714	CP077977.1
Pseudomonas aeruginosa strain PaLo17 chromosome, complete genome	Pseudomonas ...	34.2	98.6	100%	97	100.00%	6225254	CP075833.1
Pseudomonas aeruginosa strain Ned_5_BIM chromosome, complete genome	Pseudomonas ...	34.2	98.6	100%	97	100.00%	6291135	CP127122.1
Pseudomonas aeruginosa strain Pa021 chromosome, complete genome	Pseudomonas ...	34.2	98.6	100%	97	100.00%	6334617	CP177307.1
Pseudomonas aeruginosa strain strain KUD2 chromosome, complete genome	Pseudomonas ...	34.2	98.6	100%	97	100.00%	6941065	CP138577.1
Boeremia exigua uncharacterized protein (C7974DRAFT_418989), partial mRNA	Boeremia exigua	34.2	34.2	100%	97	100.00%	1254	XM_046144189.1
Pseudomonas aeruginosa PA99 chromosome, complete genome	Pseudomonas ...	34.2	98.6	100%	97	100.00%	6946480	CP042967.1
Pseudomonas aeruginosa strain PaLo33 chromosome, complete genome	Pseudomonas ...	34.2	98.6	100%	97	100.00%	6793049	CP075851.1
Pseudomonas aeruginosa strain S33 chromosome	Pseudomonas ...	34.2	98.6	100%	97	100.00%	6911712	CP142446.1
Pseudomonas aeruginosa strain A31771 chromosome	Pseudomonas ...	34.2	98.6	100%	97	100.00%	7022620	CP166029.1
Pseudomonas aeruginosa strain F052 chromosome, complete genome	Pseudomonas ...	34.2	98.6	100%	97	100.00%	6578986	CP115227.1
MAG: Spirochaetia bacterium isolate 029c9a96-c795-42b2-9c0d-59f5314b817a genome assembly, chro...	Spirochaetia ba...	34.2	34.2	100%	97	100.00%	6438990	OY759747.1
Pseudomonas aeruginosa strain 12939 chromosome, complete genome	Pseudomonas ...	34.2	98.6	100%	97	100.00%	6621378	CP024477.1
Pseudomonas aeruginosa strain R05 chromosome, complete genome	Pseudomonas ...	34.2	98.6	100%	97	100.00%	6255746	CP069324.1
Pseudomonas aeruginosa CRPA14_6.2 DNA, complete genome	Pseudomonas ...	34.2	98.6	100%	97	100.00%	6948702	AP040349.1
Pseudomonas aeruginosa strain DVT423 chromosome, complete genome	Pseudomonas ...	34.2	98.6	100%	97	100.00%	6716245	CP050326.1

Alignment results showed that over 99% of BLAST hits for the LB primer exhibited homology exclusively with Pseudomonas aeruginosa when compared against the entire NCBI database.

Alignment conclusions:

Absolutely conserved sequences: B3, FIP, BIP, LF, LB

Non-conserved sequence: F3

Among the primer set, only F3 is a non-absolutely conserved sequence. The roles of F3 and B3 are to provide chain displacement anchoring, while the specificity of the amplification product is primarily determined by FIP and BIP. Therefore, the specificity of this primer set meets the required criteria.
